# The Impact of pH Value on Corrosion Behavior of 316L, 2507 and TA2 Alloys

**DOI:** 10.3390/ma19132863

**Published:** 2026-07-04

**Authors:** Yongle Kou, Xiaoyu Liu, Qinglin Li

**Affiliations:** 1 School of Materials Science and Engineering, Northwestern Polytechnical University, Xi’an 710072, China; 2 China National Heavy Machinery Research Institute Co., Ltd., Xi’an 710018, China; 3 College of Materials Engineering Technology and Application, Puyang Petrochemical Vocational and Technical College, Puyang 457000, China; 18602982886@163.com; 4 School of Materials Science and Engineering, Lanzhou University of Technology, Lanzhou 730050, China; liql301@mail.nwpu.edu.cn

**Keywords:** ammonia-based desulfurization, pitting corrosion, pH, passive film, 316L stainless steel, 2507 duplex stainless steel, TA2 commercially pure titanium

## Abstract

The corrosion resistance of metallic materials is closely related to their service environment. In ammonia-based desulfurization post-treatment systems, 316L stainless steel, 2507 duplex stainless steel, and TA2 commercially pure titanium are widely used as candidate materials for key components such as desulfurization heat exchangers. In this study, the pitting corrosion behavior of 316L, 2507, and TA2 was investigated in simulated ammonia desulfurization post-treatment solutions with different pH. The results show that increasing solution acidity leads to a decrease in the capacitive arc radius and polarization resistance, while the donor concentration and pitting susceptibility of the three materials increase. Under the same pH condition, TA2 exhibits the highest stability and corrosion resistance, followed by 2507, whereas 316L shows the poorest corrosion resistance. The composition of the TA2 passivation film (TiO_2_) does not change as the pH of the simulated solution is modified. With increasing solution acidity, the relative XPS peak-area fraction of TiO_2_ in TA2 increases, indicating that TiO_2_ remains the dominant component of the passive film. In contrast, the relative contents of Cr- and Mo-containing oxides/hydroxides in 316L and 2507 decrease, and MoO_3_ is replaced by MoO_2_ under acidic conditions. These changes suggest weakened passive-film stability and reduced protection of the substrate.

## 1. Introduction

Desulfurization technology was rapidly developed and widely applied in industrial production in the 1970s. Many desulfurization strategies have been investigated, and among these, about a dozen flue gas desulfurization (FGD) technologies have been economically developed to maturity. One such technology is ammonia desulfurization, which is used to convert sulfur dioxide into ammonia sulfate fertilizer with excellent performance. At present, ammonia desulfurization is considered to be an increasingly mature desulfurization process for industrial applications due to its advantages of fast desulfurization speed, high efficiency, low energy consumption, short shutdown time, convenient operation, and high economic value of desulfurization products [1,2,3,4].

The efficiency of the ammonia desulfurization processes is remarkable. However, in actual desulfurization applications, ammonia desulfurization faces the problems of bad environments, the need for equipment with strong anti-corrosion properties, and difficult maintenance after equipment corrosion. The corrosion of desulfurization equipment directly affects desulfurization efficiency, the operational lifetime of desulfurization systems, and safety. Thus, the corrosion of ammonia desulfurization systems requires greater attention. A particularly prominent corrosion problem in ammonia desulfurization units is the corrosion of the heat exchanger in the post-treatment liquid. This heat exchanger is always exposed to the high-temperature acidic post-treatment liquid, which contains a high chloride ion concentration. The composition of this post-treatment liquid is complex, and as this liquid becomes more concentrated, the chloride ions in the slurry are continuously enriched, which leads to the heat exchanger undergoing more serious pitting corrosion [5,6].

The implementation of FGD strategies is a crucial measure for effectively regulating industrial SO_2_ emissions. Thus, the FGD industry has rapidly developed in recent years [7]. However, the working environment of desulfurization equipment is complex, contains many coexisting components, and experiences significant temperature fluctuations. This leads to the serious corrosion of desulfurization equipment [8,9,10]. This can lead to severe corrosion and consequently reduce the service life of the equipment [11]. Low pH, which indicates lower OH^−^ content, may promote pitting corrosion, so pH is a particularly notable environmental factor [12]. Competition between Cl^−^ and OH^−^ ions leads to a decline in hydroxide content under acidic conditions, which enhances the corrosion effects of the chloride ions. Moreover, the chloride formed by the chloride ions will continue to reduce the pH of the passivation film on the surface of a metal material. This diminishes the stability of the passivation film, rendering it susceptible to pitting corrosion [13,14,15]. Therefore, in this work, the pitting corrosion risk of heat exchanger equipment was studied in a simulated working environment, and the optimal material in this environment was determined by evaluating the corrosion of 316L stainless steel, 2507 duplex stainless steel, and TA2 in the post-treatment section of an ammonia desulfurization process under different pH.

The passivation range of 316L stainless steel has been reported to proportionally expand as the pH in a boric acid solution increases. With increasing pH, the passivation range widens and the pitting potential increases, which enhances the corrosion resistance of the stainless steel [16].

Numerous experimental results have shown that the critical pitting temperature can serve as an important parameter for evaluating the corrosion resistance of stainless steels [17,18]. In general, a higher critical pitting temperature indicates that the passive film can remain stable over a wider temperature range, suggesting stronger resistance to pit initiation and propagation. In addition to temperature-related evaluation parameters, alloying elements can also influence the corrosion resistance of stainless steels by modifying the composition and stability of the passive film. For instance, Sb addition has been reported to promote the formation of a protective Sb_2_O_5_ oxide film on the steel surface, thereby reducing the corrosion rate, with 0.1 wt.% Sb steel showing relatively superior corrosion resistance [19]. Moreover, the synergistic addition of Cu, Sb, and Ni can increase the corrosion potential and enhance the corrosion resistance of low-alloy steels, which is closely related to the existence form of Cu in the corrosion product layer [20]. In addition to low-alloy steels, stainless steels have also been widely investigated as corrosion-resistant materials for desulfurization systems. Wang et al. [21] compared the crevice corrosion behavior of 316, 904L, 254SMO, and 2507 stainless steels in high-temperature desulfurization solutions and found that 254SMO and 2507 exhibited better resistance to crevice corrosion, whereas 316 and 904L suffered more severe crevice corrosion.

The pitting resistance of 2205 duplex stainless steel at 20 °C, 50 °C, and 80 °C accelerates the dissolution of anodic metal and promotes pitting corrosion as the temperature increases [22]. At present, there is no more research on the effect of pH on the pitting corrosion of 2507 duplex stainless steel, but by studying the pitting corrosion behavior of several super stainless steels in simulated flue gas desulfurization (FGD) environments, it is found that 2507 stainless steel is a suitable material for FGD equipment [23]. Therefore, the use of 2507 stainless steel for desulfurization applications under different environmental conditions deserves further study.

The corrosion resistance of industrial TA2 pure titanium in simulated desulphurization wet flue gas is related to the pH, temperature, and fluoride content of the condensate. The corrosion resistance of TA2 pure titanium exhibits a positive correlation with the pH. With increasing condensate temperature, the corrosion current density increases, which affects the dissolution of the oxide film on the TA2 surface. Better corrosion resistance is exhibited at lower condensate temperatures. However, as the condensate temperature increases, the oxide film of TA2 is destroyed, the dissolution rate increases, and corrosion resistance deteriorates. In addition, the hydrogen fluoride present in the condensate significantly compromises the integrity of the TA2 passivation film and induces material corrosion [24]. The novelty of this work lies in establishing a comparative corrosion-resistance sequence for 316L, 2507, and TA2 under identical pH-controlled simulated FGD post-treatment conditions, and in revealing how acidification affects passive-film stability through changes in donor concentration, oxide/hydroxide composition, and Mo-containing species.

In this work, the pitting behavior of 316L stainless steel, 2507 duplex stainless steel, and TA2 pure titanium was investigated under a simulated ammonia desulfurization post-treatment environment at different pH. The effect of different pH on the pitting behavior of these three metal materials was studied. The polarization curves, AC impedances, Mott–Schottky curves, corrosion morphologies, and passivation film compositions of these metal materials were evaluated in the simulated solution at pH of 4.6, 5.6, 6.6, and 7.6. This combined analysis provides a clearer basis for material selection in ammonia-based desulfurization heat-exchanger systems.

## 2. Experimental Materials and Methods

### 2.1. Experimental Methodology

Three metallic materials, namely 316L stainless steel, 2507 duplex stainless steel, and TA2 commercially pure titanium, were selected as the experimental materials. These materials are commonly used as candidate materials for key components in ammonia-based desulfurization post-treatment systems, such as desulfurization heat exchangers. The chemical compositions of the three materials are listed in Table 1.

The samples were cut into specimens with dimensions of 10 mm × 10 mm. Before electrochemical testing, the working surfaces of all specimens were successively ground with SiC abrasive papers from 400# to 2000#, followed by polishing with diamond paste to obtain a smooth surface. The polished samples were ultrasonically cleaned in ethanol and deionized water, and then dried with cold air. For electrochemical measurements, each specimen was sealed with epoxy resin, leaving an exposed working area of 1 cm^2^.

### 2.2. Preparation of Simulated Ammonia Desulfurization Post-Treatment Solutions

The simulated ammonia-based desulfurization post-treatment solutions were prepared using analytical-grade reagents and deionized water. In the pH-dependent corrosion tests, the concentrations of Cl^−^ and SO_4_^2−^ in the final solutions were fixed at 7.0 wt.% and 10.0 wt.%, respectively, as shown in Table 2. The pH of the solutions were adjusted to 4.6, 5.6, 6.6, and 7.6 using dilute sulfuric acid or ammonia solution. After pH adjustment, the solutions were magnetically stirred to ensure chemical homogeneity, and the pH was measured again before electrochemical testing.

### 2.3. Electrochemical Measurements

All electrochemical measurements, including potentiodynamic polarization, electrochemical impedance spectroscopy (EIS), and Mott–Schottky tests, were performed in the simulated solutions at 65 ± 1 °C using a thermostatic water bath. The electrochemical measurements were carried out using a CS310M electrochemical workstation (Wuhan Corrtest Instruments Co., Ltd., Wuhan, China) with a conventional three-electrode system. A platinum sheet was used as the counter electrode, a saturated calomel electrode (SCE) was used as the reference electrode, and the specimen with an exposed area of 1 cm^2^ was used as the working electrode. The solutions were naturally aerated during the tests and were not deaerated. All potentials reported in this work are given with respect to the SCE, and no additional reference-electrode temperature correction was applied.

For potentiodynamic polarization tests, the specimen was first cathodically polarized at −1.0 V vs. SCE for 300 s. The polarization scan was then started from −1.0 V vs. SCE and swept in the anodic direction to 1.6 V vs. SCE at a scan rate of 1 mV/s. For 316L and 2507, the scan was reversed when the current reached 1 mA and was then swept back to the initial potential at the same scan rate. For TA2, only the forward anodic scan was performed because no obvious pitting loop appeared within the tested potential range. Each condition was tested at least three times to ensure reproducibility, and representative curves are shown in the manuscript.

For EIS measurements, the specimen was cathodically polarized at −1.0 V vs. SCE for 300 s, followed by stabilization at the open-circuit potential for 1 h. The EIS measurements were performed over a frequency range from 10^5^ to 10^−2^ Hz with a sinusoidal perturbation amplitude of 10 mV.

For Mott–Schottky measurements, the specimen was also cathodically polarized at −1.0 V vs. SCE for 300 s and then stabilized at the open-circuit potential for 1 h. The Mott–Schottky tests were conducted at a frequency of 1000 Hz with an AC amplitude of 10 mV. The potential was scanned from 0 to −1.0 V vs. SCE.

### 2.4. XPS Analysis

X-ray photoelectron spectroscopy was performed using an X-ray photoelectron spectrometer (ULVAC-PHI, Chigasaki, Japan) with an Al Kα X-ray source to analyze the chemical composition and valence states of the passive films formed on the surfaces of 316L, 2507, and TA2 after electrochemical testing in simulated ammonia-based desulfurization post-treatment solutions with different pH. For XPS analysis, the specimens were immersed in the pH 7.6 or pH 4.6 simulated solutions at 65 ± 1 °C for 24 h. The binding energies were calibrated using the C 1s peak at 284.8 eV. The XPS spectra were fitted after background subtraction using a Shirley background. Peak deconvolution was performed by constraining the peak positions, full widths at half maximum, and spin–orbit splitting according to reference XPS data and relevant literature values.

### 2.5. Corrosion Morphology Observation

A Gemini SEM 300 field emission scanning electron microscope (SEM, Carl Zeiss, Oberkochen, Germany) was employed to observe the surface corrosion morphologies of 316L, 2507, and TA2 after electrochemical testing. Before observation, the samples were gently cleaned with deionized water and ethanol to remove residual solution from the surface, and then dried with cold air. The specimens for SEM observation were obtained after electrochemical testing in the simulated solutions at 65 ± 1 °C.

## 3. Results and Discussion

### 3.1. Polarization Curve Analysis

**Figure 1:**
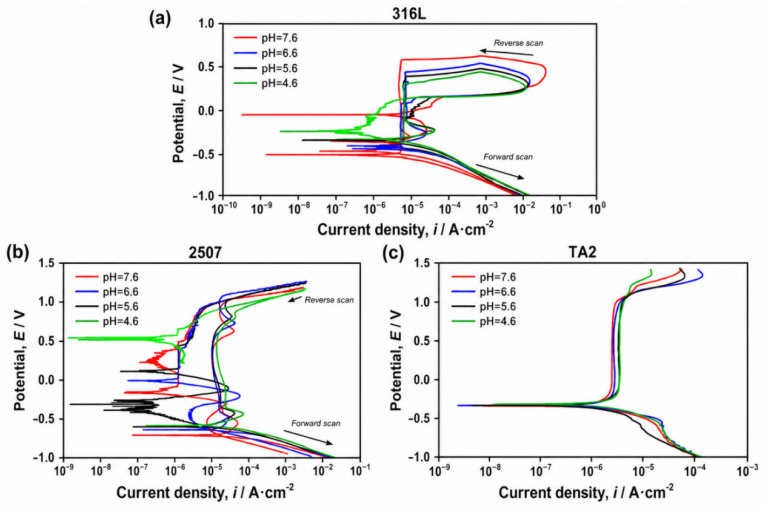
Cyclic polarization curves of (**a**) 316L stainless steel, (**b**) 2507 duplex stainless steel, and (**c**) TA2 commercially pure titanium in simulated ammonia-based desulfurization post-treatment solutions containing 7.0 wt.% Cl^−^ and 10.0 wt.% SO_4_^2−^ at different pH and 65 °C.

To improve the readability of the cyclic polarization curves, Figure 1 has been redrawn with enlarged subfigures, clearer line widths, and arrows indicating the forward and reverse scanning directions. The cyclic polarization curves of 316L, 2507, and TA2 in the simulated solutions containing 7.0 wt.% Cl^−^ and 10.0 wt.% SO_4_^2−^ at different pH and 65 °C are shown in Figure 1. The cyclic polarization curves of 316L in solutions with different pH are depicted in Figure 1a. These cyclic polarization curves have similar shapes and they each show a hysteresis loop, indicating the formation of a stable passivation zone. With decreasing pH, the passivation range decreases and the metal pitting sensitivity increases [25]. The pitting potentials at pH 7.6, 6.6, 5.6, and 4.6 are 0.321, 0.177, 0.122, and 0.027 V, respectively. Lowering the solution pH leads to a gradual increase in the passive current density I_p_ of 316L, which compromises the stability of its passivation film and diminishes its resistance to pitting.

The cyclic polarization curves of 2507 in the solutions with different pH are shown in Figure 1b. The curves exhibit similar passivation characteristics, but the anodic branch changes with solution pH. Compared with the solutions at pH 7.6, 6.6, and 5.6, the curve obtained at pH 4.6 shows a higher passive current density and a lower breakdown potential, indicating that stronger acidity promotes passive-film destabilization and increases the susceptibility of 2507 to localized corrosion. This indicates that as the pH decreases, the pitting potential of 2507 also gradually decreases. Notably, 2507 demonstrates optimal pitting resistance at a pH of 7.6. When the pH decreases, the passive current density I_p_ increases, passivation film formation becomes more difficult, and the protective performance of this metal decreases. However, at a pH of 7.6, the cyclic polarization curve returns to its original shape, and pitting corrosion is not observed. Table 3 shows that the protective potential E_p_ of the 2507 duplex stainless steel does not significantly change with the solution pH. Therefore, the ability of this metal material to repair its passivation film after film rupture is not significantly affected by the solution pH.

The cyclic polarization curves of TA2 in solutions with different pH are shown in Figure 1c. No obvious activation peak is observed, indicating that TA2 maintains a stable self-passivation state in the simulated solutions. Since the Cl^−^ concentration was kept constant, the change in the polarization behavior should be attributed mainly to the variation in solution pH rather than to chloride concentration. As the pH decreases, the breakdown potential of TA2 decreases and the passive current density increases, indicating that stronger acidity weakens the stability of the passive film and increases the susceptibility of TA2 to localized corrosion.

Table 3 shows that changing the solution pH does not significantly affect the self-corrosion potential of TA2. However, with decreasing pH, the pitting potential of TA2 decreases from 1.271 V to 1.162 V while the passive current density increases. These results indicate that a lower pH enhances the corrosion tendency and reduces the corrosion resistance of TA2.

Therefore, 316L, 2507, and TA2 exhibit favorable corrosion resistance in solutions with varying pH. As the solution pH decreases, the pitting potentials of these three metals decrease, while their passive current densities increase. Under the same conditions, TA2 shows the best pitting resistance. With decreasing pH, the protective efficacy of the passivation films diminishes, pitting sensitivity increases, and pitting resistance decreases for all three metals.

### 3.2. Electrochemical Impedance Analysis

**Figure 2:**
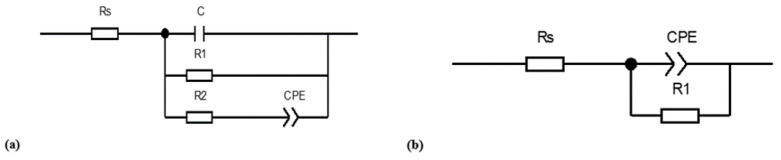
Equivalent circuit diagram: (**a**) equivalent circuit diagram of 316L and 2507; (**b**) equivalent circuit diagram of TA2.

**Figure 3:**
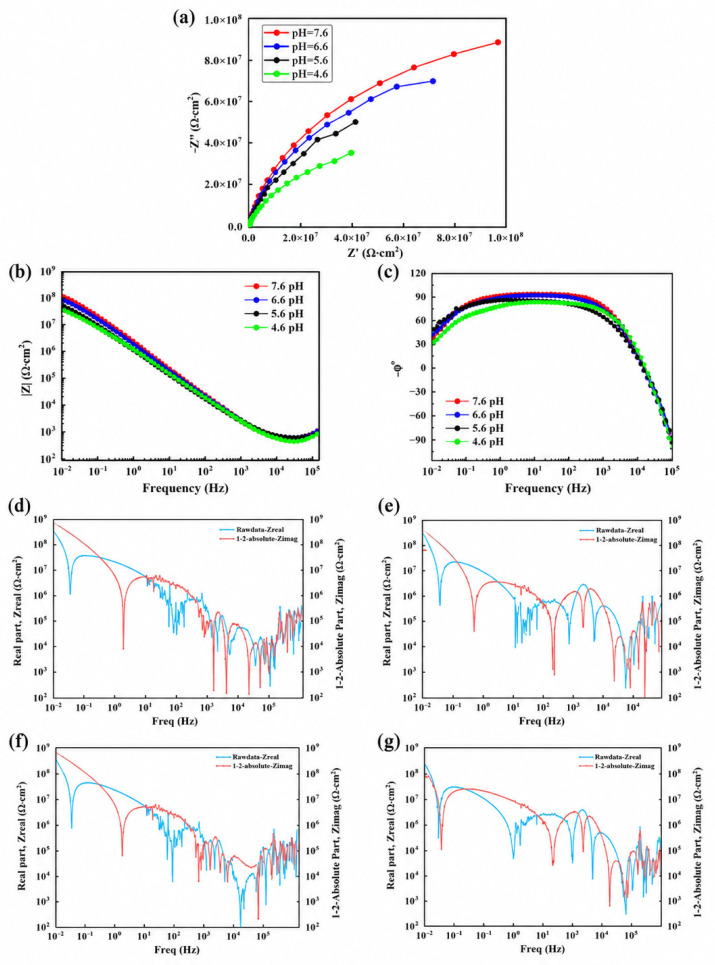
AC impedance spectra of 316L steel immersed in solutions with different pH: (**a**) Nyquist plot, (**b**) Bode modulus plot, (**c**) phase angle plot, and (**d**–**g**) 7.6–4.6 ph residual error plot.

**Figure 4:**
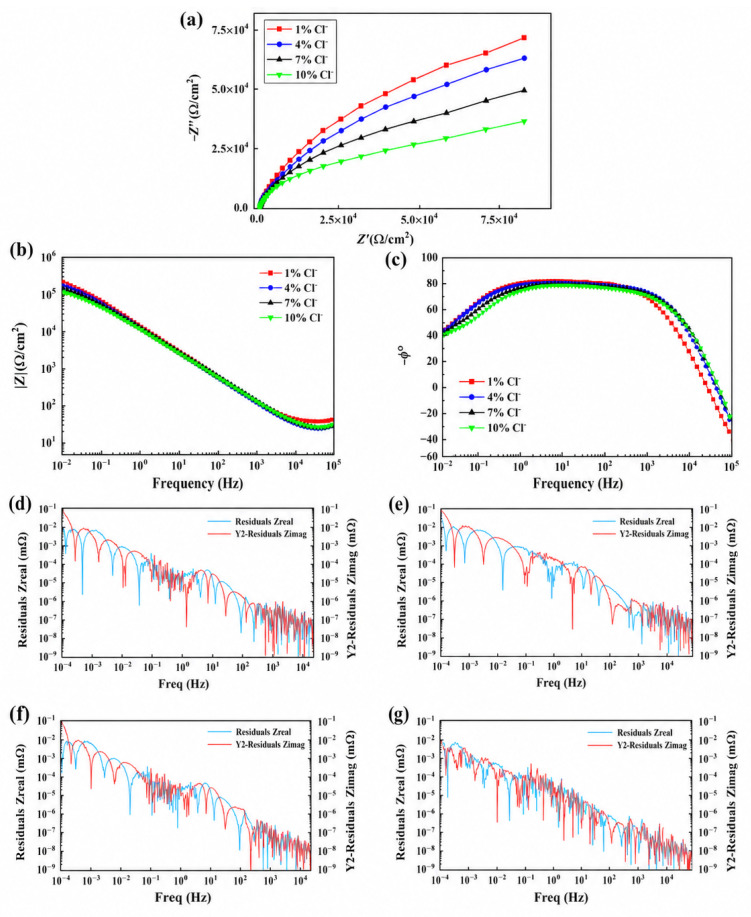
2507 AC impedance spectra in different pH solutions: (**a**) Nyquist diagram; (**b**) Bode and (**c**) phase angle diagram and (**d**–**g**) 7.6–4.9 ph residual error plot.

**Figure 5:**
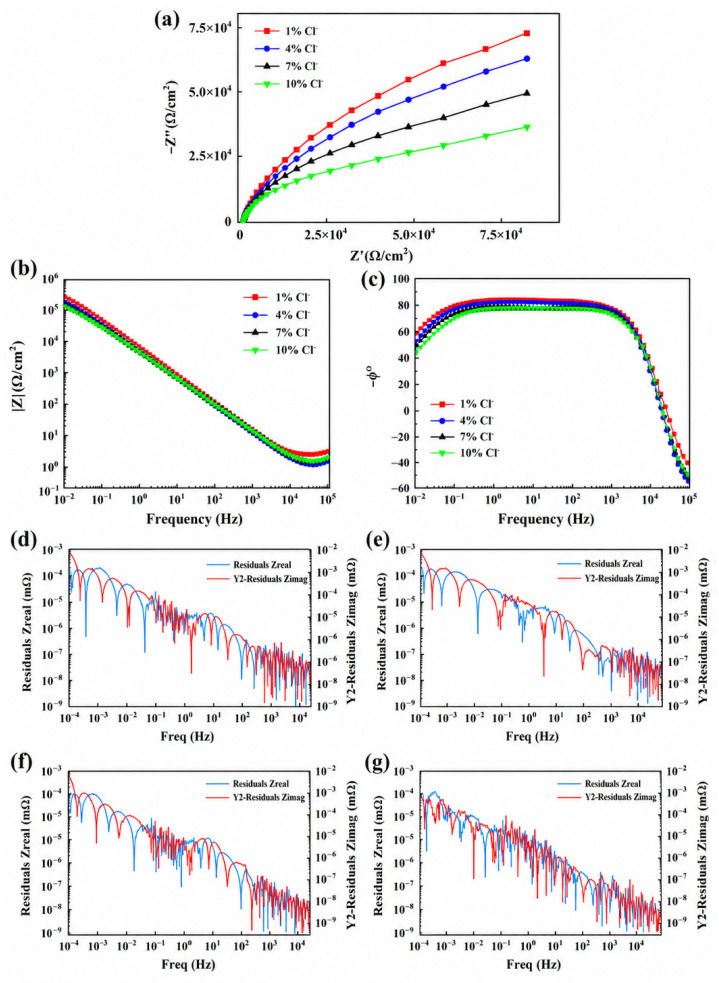
AC impedance spectra of TA2 in different pH solutions: (**a**) Nyquist diagram; (**b**) Bode and (**c**) phase angle diagram and (**d**–**g**) 7.6–4.9 ph residual error plot.

Figure 2 shows the equivalent circuit diagram used for fitting the ElS data. As shown in Figure 2a, the fitting equivalent circuit of 316L and 2507 is R_P_ = R_1_R_2_/(R_1_ + R_2_), and as shown in Figure 2b, the fitted equivalent circuit of TA2 is R_P_ = R_1_. The electrochemical impedance spectra of these three metal materials were fitted by their equivalent circuits, and the fitting data are shown in Table 4. The 316L, 2507, and TA2 samples were polarized at −1 V (vs. SCE) for 300 s prior to electrochemical impedance analysis and then soaked for 1 h after the stabilization of the open circuit potential. Electrochemical impedance spectra of the three samples in simulated solutions with different pH (7.6, 6.6, 5.6, and 4.6) were obtained to evaluate the stability of their metal passivation films, as displayed in Figure 3, Figure 4 and Figure 5. 

In the equivalent circuits, Rs represents the solution resistance between the working electrode and the reference electrode. For 316L and 2507, R1 is related to the charge-transfer resistance through the passive film or at the film/metal interface, while R2 represents the resistance associated with the defective outer corrosion-product layer and localized corrosion paths. C represents the capacitance of the compact passive-film layer. The constant phase element (CPE) was introduced to account for non-ideal capacitive behavior caused by surface roughness, passive-film heterogeneity, and localized corrosion damage. The CPE impedance can be expressed as ZCPE = [Y0(jω)n] − 1 where Y0 is the CPE admittance parameter, ω is the angular frequency, j is the imaginary unit, and n is the dispersion exponent. When n approaches 1, the CPE behaves close to an ideal capacitor; a lower n value indicates stronger surface heterogeneity and poorer film compactness. For TA2, a simpler Rs-(R1||CPE) circuit was used because TA2 forms a relatively dense TiO2-dominated passive film and its impedance response is mainly controlled by one dominant barrier-film process.

The Nyquist plots of 316L, 2507, and TA2 shown in Figure 3, Figure 4 and Figure 5 have similar shapes and are characterized by a single capacitive reactance arc. Moreover, the radius of the capacitive reactance arc decreases with decreasing pH, indicating that corrosion resistance decreases and pitting tendency increases with increasing solution acidity. Notably, when the pH is 7.6, the largest capacitive arc radius is displayed for all three metal materials, suggesting excellent passivation film stability and enhanced corrosion resistance at this pH. Moreover, under the same pH conditions, TA2 shows the largest capacitive arc radius of the three metal materials, indicating that the passivation film of TA2 has the best protection ability and highest corrosion resistance.

As indicated by Table 4, 316L, 2507, and TA2 all exhibit their highest polarization resistance R_P_ in the simulated solution with a pH of 7.6. With increasing acidity, the polarization resistance of these three metal materials decreases, and TA2 exhibits the highest polarization resistance. When the pH decreases from 6.6 to 5.6, the R_P_ value of TA2 decreases by an order of magnitude. However, TA2 still has a higher R_P_ value than that of the other two materials at pH 5.6. Therefore, the corrosion rates of all three metal materials increase as the pH decreases, indicating that the protective effects of their passivation films weaken with increasing acidity. Compared with the other two metals, TA2 has the strongest corrosion resistance.

The charge transfer resistance R1 of the three metal materials gradually decreases with decreasing simulated solution pH. This indicates that in the reaction process, lowering the pH (i.e., increasing the acidity) simplifies the charge transfer process compared to that in solutions with higher pH. Consequently, the pitting tendency increases and the corrosion rate increases when the pH decreases. In addition, the dispersion constant n decreases with decreasing pH for all three metal materials. Thus, with increasing H^+^ concentration in the solution, the compactness of the passivation film on the metal surface decreases and the corrosion resistance weakens. In addition, the value of Y_0_ increases as the pH of the simulated solution decreases. Consequently, the impedance modulus (1/Y_0_) decreases with decreasing solution pH. This demonstrates that the stability of the passivation film declines as the solution pH decreases for all three metal materials. Consequently, the ability of the passivation film to protect the matrix is reduced, which negatively affects the corrosion resistance of the materials. At the same pH, TA2 has the lowest Y_0_ value (i.e., the highest reciprocal-impedance modulus) of the three metal materials. Thus, under the same conditions, the passivation film of TA2 has the best stability and the strongest protective effect on the matrix.

In summary, the corrosion resistances of 316L, 2507, and TA2 in simulated solutions with different pH can be ordered as follows: pH = 7.6 > pH = 6.6 > pH = 5.6 > pH = 4.6. Moreover, under the same pH conditions, TA2 has the strongest corrosion resistance, which is in agreement with the cyclic polarization curve measurements conducted under these experimental conditions.

### 3.3. Mott–Schottky Curve Analysis

**Figure 6:**
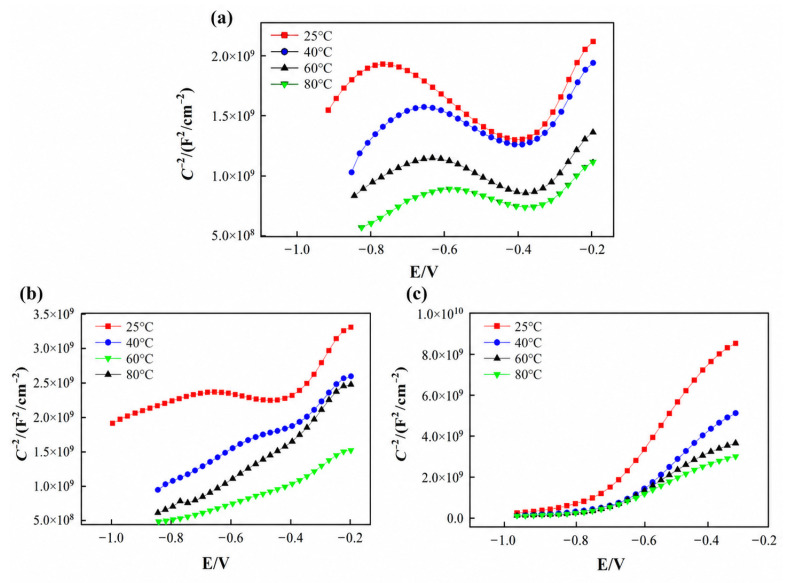
Mott–Schottky curve in different pH solutions: (**a**) 316L, (**b**) 2507, (**c**) TA2.

The 316L, 2507, and TA2 samples were polarized at −1 V (vs. SCE) for 300 s in simulated solutions with pH of 7.6, 6.6, 5.6, and 4.6, then soaked for 1 h after the open circuit potential was stabilized. Mott–Schottky curves were obtained to investigate the semiconductor properties of the passivation films as well as the presence of point defects in these films. The obtained curves are depicted in Figure 6.

The Mott–Schottky curves of 316L displayed in Figure 6a do not show any discernable changes in shape or position when the solution pH is changed. As the scanning potential increases, three linear intervals can be observed in these curves. The positive and negative values of the interval slopes indicate that the 316L passivation film exhibits n-type, p-type, and n-type semiconductor properties. Thus, the passivation film semiconductor properties change twice. According to the passivation interval corresponding to the measured polarization curves, the passivation films of 316L under different solution pH are n-type semiconductors. Taking the simulated solution with a pH of 7.6 as an example, the obtained donor concentration N_D_ of 316L is 2.851 × 10^21^ a/cm^3^, which is lower than that of this metal under other pH. Thus, at a pH of 7.6, 316L exhibits the lowest point defect content, the most difficult charge transfer, the largest passivation film thickness, and the strongest resistance to pitting corrosion. With decreasing pH, the acidity increases, the donor concentration increases, the stability of the passivation film weakens, and the pitting tendency increases.

The Mott–Schottky curves of 2507 in simulated solutions with different pH are illustrated in Figure 6b. The curves obtained at pH 7.6 and 6.6 exhibit similar features, whereas those obtained at pH 5.6 and 4.6 show another similar trend. This indicates that the semiconductor behavior of the passive film on 2507 changes with increasing solution acidity. At pH 7.6 and 6.6, the Mott–Schottky plots show a negative slope in the potential range from −1.0 to −0.6 V, suggesting p-type semiconductor behavior in this region. When the potential is higher than −0.6 V, the slope becomes positive, indicating a transition to n-type semiconductor behavior. In contrast, at pH 5.6 and 4.6, the passive film mainly exhibits n-type semiconductor characteristics over the analyzed potential range.

This difference may be attributed to the decreased stability and increased defect density of the passive film under more acidic conditions. At relatively high pH, the Cr- and Mo-containing oxide/hydroxide species in the passive film are more stable, resulting in a more compact film structure and lower defect concentration. As the pH decreases, the increased H^+^ concentration and aggressive Cl environment promote the dissolution of protective species and generate more donor-type defects, such as oxygen vacancies, in the passive film. As a result, the donor concentration increases and the n-type semiconductor response becomes more pronounced. As shown in Table 5, 2507 exhibits the lowest donor concentration and the strongest pitting resistance at pH 7.6, whereas the highest donor concentration is observed at pH 4.6, indicating reduced passive-film stability and increased susceptibility to pitting corrosion.

The Mott–Schottky curves of TA2 in simulated solutions with different pH are depicted in Figure 6c. These curves exhibit consistent behavior. Within the scanning potential range of −1.0 to 0.2 V, the measured Mott–Schottky curves obtained in the simulated solutions at various temperatures display a positive slope, indicating that the passivation film of TA2 demonstrates n-type semiconductor characteristics under different pH conditions. Within this scanning potential range, the obtained Mott–Schottky curves show two potential intervals: a nonlinear interval and a linear interval. At pH of 7.6, 6.6, 5.6, and 4.6, N_D_ values of 3.343 × 10^20^ a/cm^3^, 3.509 × 10^20^ a/cm^3^, 3.689 × 10^20^ a/cm^3^, and 3.838 × 10^20^ a/cm^3^ are respectively obtained, indicating that the N_D_ value increases with decreasing pH. The lowest N_D_ value and strongest pitting resistance are observed at a solution pH of 7.6. In contrast, the highest N_D_ value and largest number of point defects are obtained at a solution pH of 4.6. Under this low pH, the metal passivation film is most easily ruptured and the TA2 metal material has the weakest pitting resistance.

In summary, as the pH of the simulated solution decreases, the donor concentrations of 316L, 2507, and TA2 all increase. This indicates that the carrier density and point defect content both increase as the solution acidity increases. Consequently, charge transfer becomes more challenging, the stability of the passivation film decreases, and pitting resistance declines. At the same solution pH, TA2 demonstrates a significantly lower donor concentration compared to 2507, while 316L exhibits the highest donor concentration. Therefore, in terms of corrosion resistance, TA2 performs exceptionally well, 2507 exhibits worse performance, and 316L has the lowest corrosion resistance.

### 3.4. X-Ray Photoelectron Analysis

**Figure 7:**
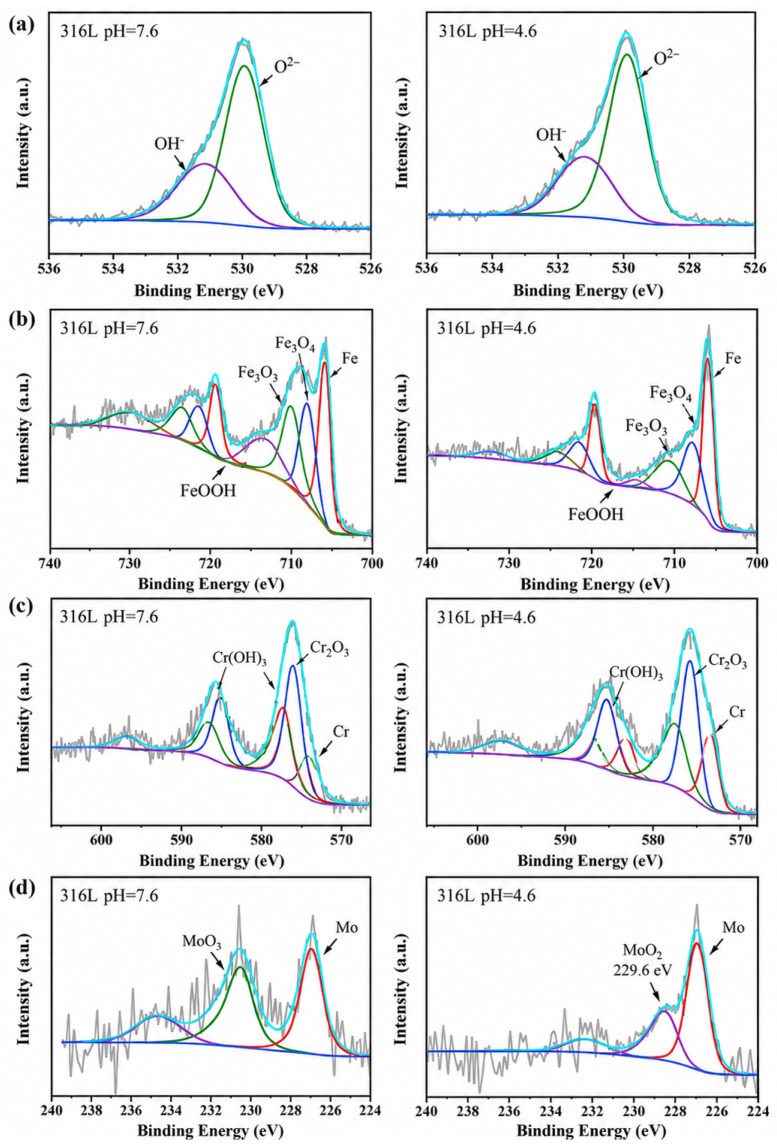
316L was immersed in a simulated solution with pH 7.6, 4.6 at 65 °C for 24 h to form a XPS pattern of passive film (**a**) O; (**b**) Fe; (**c**) Cr; (**d**) Mo.

**Figure 8:**
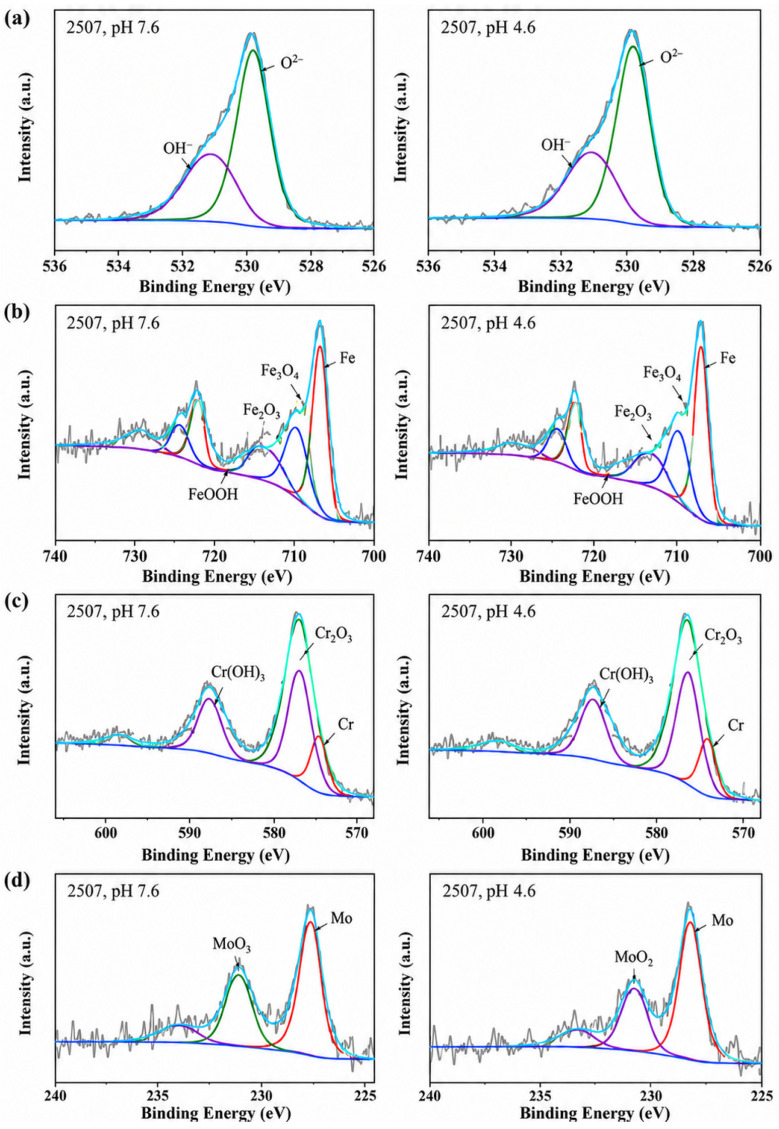
2507 the XPS pattern of passive film formed after immersion in simulated solution at 65 °C for 24 h (**a**) O; (**b**) Fe; (**c**) Cr; (**d**) Mo.

**Figure 9:**
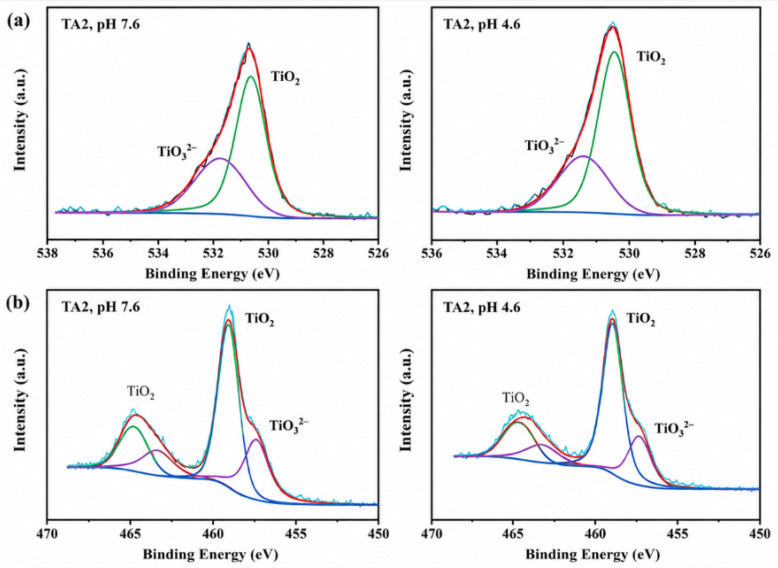
XPS pattern of passive film formed by TA2 immersed in simulated solution at 65 °C for 24 h. (**a**) O; (**b**) Ti.

Mott–Schottky analysis and the potential ranges of 316L, 2507, and TA2 in the passivation regions of the polarization curves indicate that these three metal materials are n-type semiconductors in the simulated solutions. Moreover, the semiconductor characteristics of the passivation layers remain constant even if the pH is changed. To further explore the effect of solution acidity and alkalinity on these passivation films, the metals exposed to simulated solutions with pH of 7.6 and 4.6 were evaluated by X-ray photoelectron spectroscopy (XPS). First, the 316L, 2507, and TA2 samples were immersed in pH 7.6 or pH 4.6 simulated solutions at 65 °C for 24 h. After this treatment, XPS spectra were obtained to evaluate the compositions and surface elemental valence states of the passivation films, as shown in Figure 7, Figure 8 and Figure 9. This analysis demonstrates that the passivation film compositions of 316L, 2507, and TA2 do not change after immersion in the actual post-treatment solution and simulated solution for 24 h, and changing the solution pH also does not affect passivation film composition.

As shown in Figure 7, the fitted O 1s spectra of 316L after treatment in the simulated solution at 65 °C and pH 7.6 or 4.6 show peaks ascribed to OH^−^ (531.2 eV) and O^2−^ (529.9 eV). As shown in Figure 7a and Table 6, the O 1s spectra of 316L can be deconvoluted into O^2−^ (529.9 eV) and OH^−^ (531.2 eV) components. Although the OH^−^ component appears to show a higher peak intensity at pH 4.6, the semi-quantitative comparison was based on the integrated fitted peak area rather than the peak height. As the solution pH decreases from 7.6 to 4.6, the relative peak-area fraction of OH^−^ decreases from 32.94% to 30.67%, while that of O^2−^ increases from 67.06% to 69.33%. This result indicates a slight decrease in hydroxide-related species in the passive film of 316L under acidic conditions, which weakens the film stability and reduces the pitting resistance. The Fe 2p XPS spectra of 316L are shown in Figure 7b. The four fitted Fe 2p_3/2_ peaks correspond to Fe, Fe_3_O_4_, Fe_2_O_3_, and FeOOH, respectively. Figure 7c displays the fitted Cr 2p_3/2_ spectra of 316L, which show peaks related to metallic Cr element, Cr_2_O_3_, and Cr(OH)_3_. As shown in Figure 7d and Table 6, the Mo 3d_5_/_2_ spectrum of the 316L passive film formed at pH 7.6 contains Mo and MoO_3_ components, while the MoO_3_ component is replaced by MoO_2_ after exposure to the pH 4.6 solution. This result indicates that the Mo-containing species in the passive film change from Mo(VI)-related oxides to Mo(IV)-related oxides under acidic conditions. Previous studies have reported that Mo species enriched in stainless-steel passive films contribute to improved resistance against chloride-induced passivity breakdown, and Mo(VI)-containing species in the outer layer can hinder the deep penetration of Cl^−^ into the passive film [26,27]. Therefore, the disappearance of the MoO_3_ component, together with the decrease in Fe and Cr oxide/hydroxide components, suggests that the stability and self-repairing ability of the 316L passive film are weakened in the pH 4.6 solution.

According to the binding energies and relative peak areas (%) of the elemental compounds listed in Table 6, changing the simulated solution pH does not alter the composition of the 316L passivation film. However, lowering the solution pH leads to a corresponding decline in the amount of OH^−^ in the 316L passivation film. In both the pH 7.6 and pH 4.6 simulated solutions, the primary constituents of the 316L passivation film are Cr, Fe, and Mo oxides and hydroxides. As indicated by Table 6, as the acidity of the simulated solution increases, a decline in Fe_2_O_3_, FeOOH, Cr_2_O_3_, and Cr(OH)_3_ content is observed. Moreover, MoO_3_ is replaced by MoO_2_. This change indicates that the ability of the 316L passivation film to repair itself is weakened, the stability of the passivation film decreases, and the protective effect of this film on the substrate declines.

The O 1s, Fe 2p, Cr 2p, and Mo 3d XPS spectra of 2507 after immersion in pH 7.6 and 4.6 simulated solutions at 65 °C for 24 h are displayed in Figure 8. The passivation film composition of 2507 is consistent with that of 316L. As displayed in Table 7, lowering the solution pH leads to a decline in the primary components of the 2507 passivation film, and the relative peak area of Fe_3_O_4_ decreases from 37.13% to 21.2%. As shown in Table 7, when the pH decreases from 7.6 to 4.6, the relative peak area of Fe_3_O_4_ decreases from 37.13% to 21.20%, that of Fe_2_O_3_ decreases from 29.68% to 21.53%, and that of FeOOH decreases from 16.23% to 11.70%. As with 316L, lowering the solution pH causes the Mo oxide phase of 2507 to change from MoO_3_ to MoO_2_, and the oxide content decreases. The decline in Cr and Mo oxide and hydroxide content with decreasing pH indicates a corresponding decline in the stability of the 2507 passivation film. Thus, at the lower pH of 4.6, the self-repair ability of the passivation film is weaker, and the corrosion resistance of this film decreases.

The XPS spectra of TA2 obtained after soaking in the pH 4.6 and 7.6 simulated solutions at 65 °C for 24 h are displayed in Figure 9. TA2 forms a passivation film under both conditions, and this film is mainly composed of TiO_2_. Thus, the passivation film of TA2 comprises high-valence titanium (Ti^4+^). As the acidity of the simulated solution increases, the relative peak-area fraction of TiO_2_ increases from 62.05% to 70.17%, as shown in Table 8. This result indicates that the analyzed surface region of the TA2 passive film remains dominated by TiO_2_-related species. However, the relative XPS peak area alone cannot be used to determine passive-film thickness. Therefore, the good corrosion resistance of TA2 under acidic conditions is mainly supported by the electrochemical results, including its lower passive current density, larger impedance response, higher polarization resistance, and lower donor density compared with 316L and 2507.

### 3.5. Corrosion Morphology

**Figure 10:**
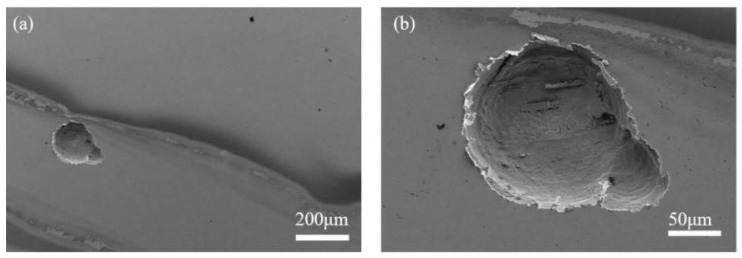
316L in pH 7.6 simulated solution, the surface corrosion morphology of the sample measured by polarization curve is: (**a**) low power; (**b**) high power.

**Figure 11:**
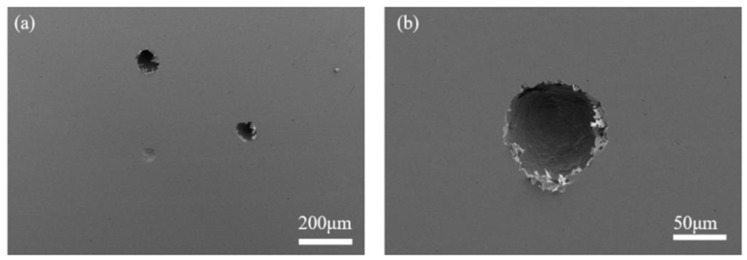
316L in pH 6.6 simulated solution, the surface corrosion morphology of the sample measured by polarization curve is: (**a**) low power; (**b**) high power.

**Figure 12:**
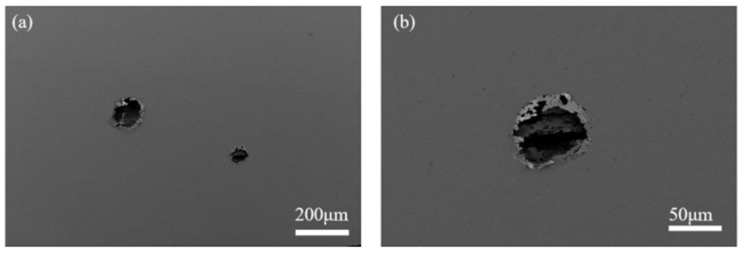
316L in pH 5.6 simulated solution, the surface corrosion morphology of the sample measured by polarization curve is as follows: (**a**) low power; (**b**) high power.

**Figure 13:**
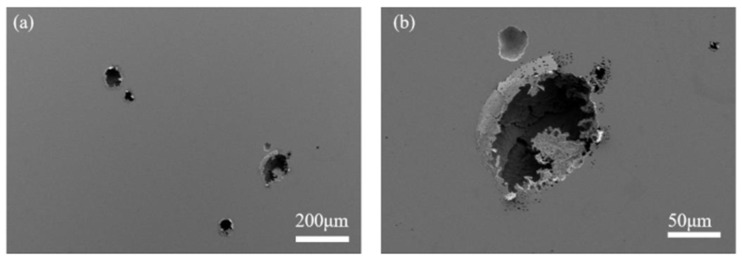
316L in pH 4.6 simulated solution, the surface corrosion morphology of the sample measured by polarization curve is: (**a**) low power; (**b**) high power.

**Figure 14:**
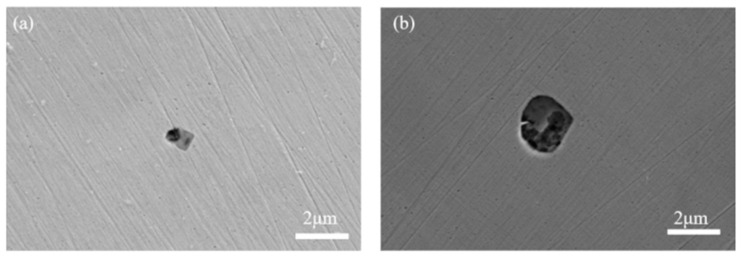
Corrosion morphology of the sample after polarization curve test, (**a**) simulated solution with pH of 2507, (**b**) simulated solution with pH of 4.6.

The corrosion behavior of 316L, 2507, and TA2 was investigated in simulated solutions with pH of 4.6, 5.6, 6.6, and 7.6. According to the obtained polarization curves, 316L shows pitting corrosion in all four simulated solutions. Meanwhile, 2507 duplex stainless steel shows pitting corrosion in the simulated solutions with pH of 5.6 and 4.6. In contrast, the passivation film of TA2 remains intact and demonstrates excellent pitting resistance in all four solutions. To further investigate the impact of pH on the corrosion morphology of these metal materials, the samples with pitting corrosion were analyzed by SEM, as presented in Figure 10, Figure 11, Figure 12, Figure 13 and Figure 14.

After immersion in the pH 7.6 simulated solution, the 316L metal surface clearly shows shallow pits with visible bottoms. As the acidity of the simulated solution increases, the pit size decreases, the pit depth increases, and a metal coating appears around the pits. When the pH decreases to 5.6, the metal coating around the pits expands, showing a lace-like shape, and the pits become deeper. When the pH decreases to 4.6, the surface pitting density, pit diameter, and metal coating area increase. Moreover, cracks and numerous small voids appear on the 316L surface, which leads to a self-catalyzed pitting corrosion effect. Thus, the corrosion rate of the metal accelerates and the sensitivity of 316L to pitting increases when the pH is decreased. After 2507 is immersed in the pH 5.6 simulated solution, a very small number of pits with a shallow depth are observed. These pits only slightly damage the surface integrity of the 2507 alloy. With increasing acidity, the depth and diameter of these pits increase, which weakens the protective effect of the passivation film on the substrate. Consequently, the pitting resistance of 2507 decreases at lower pH. Compared with the other two materials, the 2507 passivation film has a better protection effect on the matrix and stronger pitting resistance under the same acidic conditions.

### 3.6. Corrosion Mechanism

**Figure 15:**
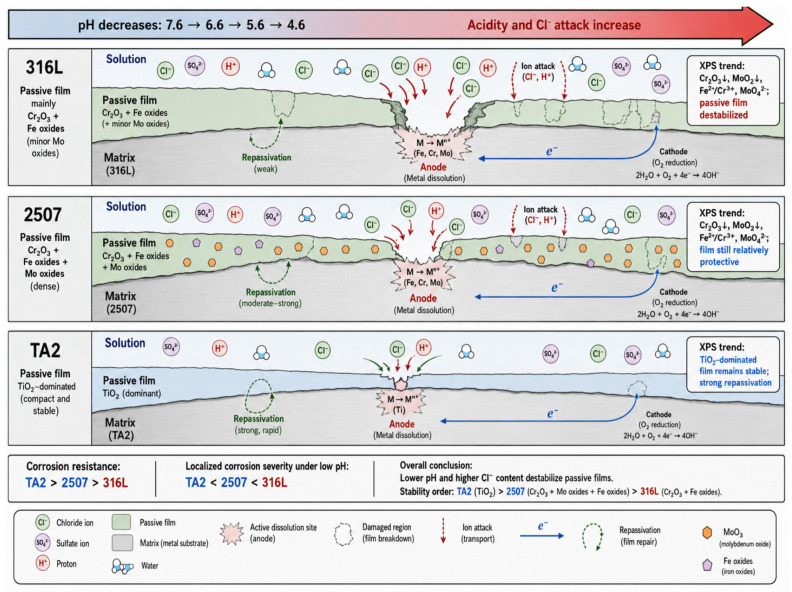
Schematic illustration of the passive film evolution and corrosion mechanism of 316L, 2507 and TA2 in dead green liquor with decreasing pH.

Figure 15 shows the corrosion mechanism diagram. Based on the electrochemical and XPS results, the corrosion mechanisms of the three materials were analyzed. The decrease in pH enhanced passive film dissolution and promoted Cl^−^-induced local breakdown. The Cr_2_O_3_/Fe oxide film on 316L showed lower stability, whereas the Cr- and Mo-containing passive film on 2507 provided stronger protection. TA2 maintained a compact TiO_2_-dominated film with rapid repassivation, which effectively inhibited corrosion propagation. Thus, the corrosion behavior of the three materials was mainly governed by passive film stability and repassivation ability.

## 4. Conclusions

In this study, the corrosion behavior of 316L stainless steel, 2507 duplex stainless steel, and TA2 commercially pure titanium was investigated in simulated ammonia-based desulfurization post-treatment solutions with different pH values using electrochemical measurements, XPS, and SEM analysis. The main conclusions are as follows:

(1) With decreasing solution pH, the pitting tendency of 316L, 2507, and TA2 increases. The pitting potential decreases, the passive current density increases, and the stability of the passive film weakens. Under the same pH condition, TA2 exhibits the best corrosion resistance, followed by 2507, while 316L shows the poorest corrosion resistance.

(2) The donor density of the passive films increases as the solution pH decreases, indicating that more donor-type defects are formed under acidic conditions. At the same pH, 316L has the highest donor density, 2507 has a lower donor density, and TA2 has the lowest donor density. This indicates that the TiO_2_-dominated passive film on TA2 has better stability and stronger resistance to localized corrosion.

(3) The composition of the TA2 passive film does not change significantly with solution pH, and the film remains mainly composed of TiO_2_-related species. Under acidic conditions, the relative peak-area fraction of TiO_2_ increases in the analyzed surface region, but this does not directly prove an increase in passive-film thickness. For 316L and 2507, the passive films are mainly composed of Cr-, Fe-, and Mo-containing oxides and hydroxides. With increasing solution acidity, the relative contents of Cr- and Mo-containing protective species decrease, and MoO_3_ is replaced by MoO_2_, indicating weakened passive-film stability.

(4) With decreasing solution pH, the pitting damage on 316L and 2507 becomes more serious, with increased pit size and pit depth. The corrosion damage of 316L is the most severe, while no obvious pitting is observed on the TA2 surface.

## Figures and Tables

**Table 1:** Main compositions of three metallic materials.

Material	C	Cr	Ni	Mn	Si	P	S	Mo	Cu	Fe	Ti
316L	0.018	17.2	12	1.4	0.35	0.01	0.006	2.1	0.3	Bal	-
2507	0.015	25.04	6.74	0.71	0.5	0.028	0.001	3.78	0.1	Bal	-
TA2	0.05	-	-	-	-	-	-	-	-	0.2	Bal

**Table 2:** Composition of the simulated solution.

Solution	Cl^−^ Concentration (wt.%)	SO_4_^2−^ Concentration (wt.%)	pH
pH-adjusted simulated solution	7.0	10.0	4.6, 5.6, 6.6, 7.6

**Table 3:** Electrochemical parameters obtained from the cyclic polarization curves of 316L stainless steel, 2507 duplex stainless steel, and TA2 commercially pure titanium in simulated ammonia-based desulfurization post-treatment solutions with different pH.

pH	7.6	6.6	5.6	4.6
316L E_b_ (V)	0.321	0.177	0.122	0.027
316L I_p_ (A/cm^2^)	3.866 × 10^−6^	6.363 × 10^−6^	6.134 × 10^−6^	6.214 × 10^−6^
316L E_corr_ (V)	−0.644	−0.593	−0.544	−0.531
2507 E_b_ (V)	1.052	1.072	1.052	0.854
2507 E_p_ (V)	―	0.933	0.964	―
2507 I_p_ (A/cm^2^)	5.938 × 10^−6^	6.019 × 10^−6^	6.045 × 10^−6^	8.142 × 10^−6^
2507 E_corr_ (V)	−0.694	−0.623	−0.583	−0.562
TA2 E_b_ (V)	1.271	1.209	1.162	1.077
TA2 E_corr_ (V)	−0.392	−0.383	−0.383	−0.367
TA2 I_p_ (A/cm^2^)	2.641 × 10^−6^	2.907 × 10^−6^	3.616 × 10^−6^	3.934 × 10^−6^

Note: E_b_ is the breakdown potential, E_p_ is the protection potential, E_corr_ is the corrosion potential, and I_p_ is the passive current density.

**Table 4:** AC impedance fitting data of three metal materials in different pH solutions.

Materials	pH Value	R_S_ (Ω·cm^2^)	C (F·cm^2^)	R_1_ (Ω·cm^2^)	R_2_ (Ω·cm^2^)	Y_0_ (Ω^−1^cm^−2^s^−n^)	n	R_p_ (Ω·cm^2^)
316L	7.6	1.266	2.994 × 10^−5^	1.996 × 10^5^	54.589	3.291 × 10^−5^	0.800	54.574
	6.6	1.299	3.017 × 10^−5^	1.754 × 10^5^	31.293	4.674 × 10^−5^	0.790	31.287
	5.6	1.337	3.025 × 10^−5^	1.600 × 10^5^	14.081	9.786 × 10^−5^	0.789	14.080
	4.6	1.385	1.797 × 10^−5^	0.938 × 10^5^	12.486	10.683 × 10^−5^	0.766	12.484
2507	7.6	1.427	2.056 × 10^−5^	3.170 × 10^5^	26.825	5.845 × 10^−5^	0.801	26.823
	6.6	1.138	2.474 × 10^−5^	2.184 × 10^5^	24.409	5.263 × 10^−5^	0.799	24.406
	5.6	1.139	1.671 × 10^−5^	2.065 × 10^5^	23.229	6.848 × 10^−5^	0.788	23.226
	4.6	1.438	1.457 × 10^−5^	1.267 × 10^5^	18.765	6.048 × 10^−5^	0.771	18.762
TA2	7.6	1.127	-	1.767 × 10^6^	-	2.158 × 10^−5^	0.945	1.767 × 10^6^
	6.6	1.194	-	1.075 × 10^6^	-	3.146 × 10^−5^	0.846	1.075 × 10^6^
	5.6	1.288	-	7.424 × 10^5^	-	3.953 × 10^−5^	0.869	7.424 × 10^5^
	4.6	1.164	-	4.460 × 10^5^	-	4.370 × 10^−5^	0.909	4.460 × 10^5^

**Table 5:** ND fitting results of donor concentrations of three metal materials in different pH solutions.

Materials	Donor Concentration	pH = 7.6	pH = 6.6	pH = 5.6	pH = 4.6
316L	ND a/cm^3^	2.851 × 10^21^	2.953 × 10^21^	3.162 × 10^21^	3.366 × 10^21^
2507	ND a/cm^3^	1.373 × 10^21^	1.932 ×10^21^	2.241 × 10^21^	2.367 × 10^21^
TA2	ND a/cm^3^	3.343 × 10^20^	3.509 × 10^20^	3.689 × 10^20^	3.838 × 10^20^

**Table 6:** 316L XPS analysis results in simulated solution at 65 °C and pH 7.6,4.6.

Spectral Peak	Species	Binding Energy (eV)	Peak Area Ratio (%)	Binding Energy (eV)	Peak Area Ratio (%)
		316L 7.6pH		316L 4.6pH	
Fe 2p_3/2_	Fe	706.5	30.95	706.4	39.13
	Fe_3_O_4_	708.6	22.29	708.2	33.27
	Fe_2_O_3_	710.5	27.29	710.8	19.43
	FeOOH	714.1	19.47	714.5	8.17
Cr 2p_3/2_	Cr	574.6	16.45	574.1	26.02
	Cr_2_O_3_	576.6	45.64	576.1	38.40
	Cr(OH)_3_	577.4	37.91	577.8	35.58
Mo 3d_5/2_	Mo	227.3	56.36	227.3	81.27
	MoO_3_	231.5	43.64	—	—
	MoO_2_	—	—	229.6	18.73
O 1s	O^2−^	529.9	67.06	529.9	69.33
	OH^−^	531.2	32.94	531.2	30.67

**Table 7:** 2507 results of XPS analysis in simulated solution at 65 °C and pH 7.6 and 4.6.

Spectral Peak	Species	Binding Energy (eV)	Peak Area Ratio (%)	Binding Energy (eV)	Peak Area Ratio (%)
		2507 7.6pH		2507 4.6pH	
Fe 2p_3/2_	Fe	706.8	16.96	706.5	45.57
	Fe_3_O_4_	709.0	37.13	708.6	21.20
	Fe_2_O_3_	710.7	29.68	710.7	21.53
	FeOOH	714.5	16.23	714.0	11.70
Cr 2p_3/2_	Cr	574.8	11.90	574.6	22.53
	Cr_2_O_3_	576.3	64.00	576.3	49.92
	Cr(OH)_3_	578.0	24.10	578.0	27.55
Mo 3d_5/2_	Mo	227.4	43.73	227.2	78.03
	MoO_3_	231.8	56.27	—	—
	MoO_2_	—	—	230.1	21.97
O 1s	O^2−^	530.0	69.66	529.9	71.54
	OH^−^	531.4	30.34	531.4	28.46

**Table 8:** Results of XPS analysis of TA2 in simulated solution at 65 °C and pH 7.6 and 4.6.

Spectral Peak	Species	Binding Energy (eV)	Peak Area Ratio (%)	Binding Energy (eV)	Peak Area Ratio (%)
		TA2 7.6pH		TA2 4.6pH	
Ti 2p	TiO_3_^2−^	457.5	37.95	457.4	29.83
	TiO_2_	459.2	62.05	459.0	70.17
O 1s	TiO_2_	530.7	63.99	530.5	69.55
	TiO_3_^2−^	531.7	36.01	531.4	32.41

## Data Availability

The original contributions presented in this study are included in the article. Further inquiries can be directed to the corresponding author.
